# Evaluative performance of TyG-ABSI versus traditional indices in relation to cardiovascular disease and mortality: evidence from the U.S. NHANES

**DOI:** 10.1186/s12933-025-02902-6

**Published:** 2025-08-21

**Authors:** Xin Zheng, Wenjing Zhang, Feng Yang, Leigang Wang, Bing Yu, Bin Liang

**Affiliations:** 1 Department of Cardiovascular Medicine, Second Hospital of Shanxi Medical University, Taiyuan, 030001 China; 2 Department of Cardiovascular Medicine, Yangquan First People’s Hospital, Yangquan, 045000 China; 3 Department of Extracorporeal Circulation, Beijing Anzhen Hospital, Capital Medical University, Beijing, 100029 China

**Keywords:** Metabolic syndrome, TyG-ABSI, Cardiovascular disease, Mortality risk, NHANES

## Abstract

**Background:**

Metabolic Syndrome (MetS) significantly increases the risk of cardiovascular disease (CVD), with central obesity and insulin resistance as major contributors. The TyG-ABSI index is a newly proposed composite measure that combines the TyG index and ABSI, aiming to assess both insulin resistance and central obesity simultaneously. Previous studies have shown that TyG-ABSI has potential in predicting cardiovascular mortality, but its applicability in MetS populations remains unclear. This study aims to explore the association between TyG-ABSI and cardiovascular events in individuals with MetS and compare its predictive value with the traditional TyG index in this specific population.

**Methods:**

Participants from the National Health and Nutrition Examination Survey (NHANES) between 2001 and 2018 were selected, with all data weighted for sample design, clustering, and stratification to ensure national representativeness. Associations between TyG-ABSI and other TyG indices with cardiovascular mortality and all-cause mortality were assessed using weighted Cox proportional hazards models; CVD prevalence was analyzed using weighted logistic regression models. Additional analyses included Kaplan–Meier survival curves and restricted cubic spline regression. Model performance was compared between TyG-ABSI, TyG, and its derived indices using ROC curves, NRI, IDI, and DCA. E-value, subgroup analyses, and competing risks models were conducted to assess robustness.

**Results:**

This study analyzed data from 12,813 individuals with metabolic syndrome in the NHANES cohort to systematically compare the performance of TyG-ABSI and other TyG-related indices in assessing CVD and mortality. The results revealed significant associations between TyG-ABSI and CVD, cardiovascular mortality, and all-cause mortality. Specifically, for each 1-unit increase in TyG-ABSI, the risk of CVD increased by 28%, cardiovascular mortality by 25%, and all-cause mortality by 28%. These associations showed a dose–response relationship in stratified analyses based on tertiles, and TyG-ABSI outperformed the traditional TyG index in overall analysis. Compared to other TyG-related indices, TyG-ABSI demonstrated superior predictive performance in metrics such as the ROC curve, NRI, and DCA. Further analyses, including competing risks models, E-value estimation, and RCS modeling, confirmed the robustness of these associations. Subgroup analyses also supported the stability of TyG-ABSI, with limited interaction effects.

**Conclusion:**

Our study highlights the value of TyG-ABSI in assessing cardiovascular disease and mortality risk in populations with MetS, providing new evidence for medical practice and public health interventions.

**Graphical abstract:**

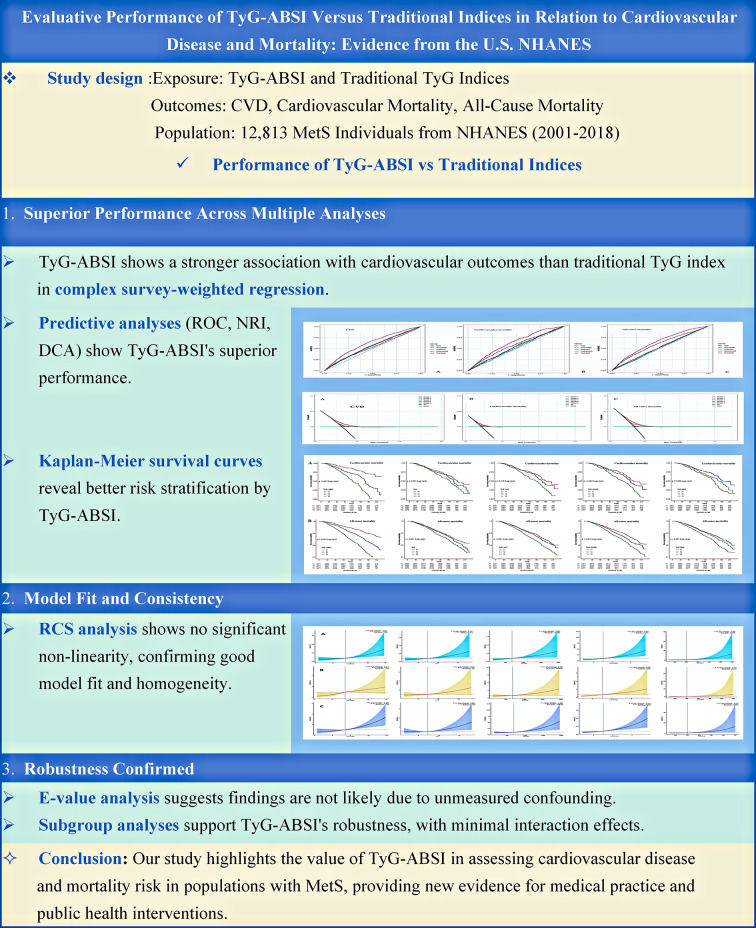

**Supplementary Information:**

The online version contains supplementary material available at 10.1186/s12933-025-02902-6.

**Research Insights**

**What is currently known about this topic?**

- MetS significantly increases the risk of cardiovascular events and mortality, highlighting the need for better assessment tools.
- The TyG index reflects insulin resistance, while ABSI better captures the pattern and risk of central obesity.
- Most existing TyG-derived indices target single components, limiting their comprehensive predictive utility.

**What is the key research question?**

- Can TyG-ABSI outperform other TyG-related indices in assessing CVD and mortality among MetS patients?

**What is new?**

- TyG-ABSI shows the highest assessment performance and strongest risk association across all statistical models.
- By combining insulin resistance and central obesity, TyG-ABSI overcomes limitations of existing single-focus indices.
- A clear linear dose–response relationship exists between TyG-ABSI and adverse outcomes in MetS populations.

**How might this study influence clinical practice?**

- TyG-ABSI may serve as a practical tool for early identification and risk stratification in MetS patients.

## Introduction

Metabolic syndrome (MetS) is defined by a cluster of metabolic abnormalities, with central obesity and insulin resistance (IR) as its defining features. These abnormalities are typically accompanied by elevated blood pressure, dyslipidemia, characterized by increased triglycerides (TG) and decreased high-density lipoprotein cholesterol (HDL-C), and hyperglycemia. Collectively, these factors substantially increase the risk of cardiovascular disease (CVD) and type 2 diabetes mellitus [1, 2]. To standardize diagnostic criteria, a harmonized definition was proposed by Alberti et al. in 2009, under which the presence of any three of the aforementioned abnormalities qualifies an individual for MetS [1]. Since the concept was first introduced in the 1980s, the prevalence of MetS has risen markedly, with a 35% increase reported in the United States by 2012 [3]. Globally, MetS is a growing public health concern, particularly in developing countries and among younger populations, affecting an estimated 20%-40% of the global population and frequently coexisting by IR [4–6].

IR plays a central role in the pathophysiology of MetS. It refers to reduced sensitivity and responsiveness of peripheral tissues to insulin, resulting in impaired glucose uptake and metabolism [7, 8]. IR promotes vascular smooth muscle cell proliferation and atherosclerosis by decreasing nitric oxide production [9] and impairs myocardial glucose utilization, contributing to metabolic disturbances and cardiac injury [10]. Given its clinical significance, the triglyceride-glucose (TyG) index has been widely adopted as a practical, low-cost, and sensitive surrogate marker of IR [11, 12]. Previous studies have shown a strong association between the TyG index and MetS, CVD, and adverse health outcomes [13].

To enhance predictive performance, researchers have developed a series of TyG-derived indices by combining the TyG index with obesity-related parameters, such as body mass index (TyG-BMI), waist circumference (TyG-WC), and waist-to-height ratio (TyG-WHtR) [14, 15]. These indices have shown utility in assessing cardiometabolic risk. However, they are generally limited to a single dimension, such as overall adiposity or IR, and often fail to fully capture the role of central obesity, which is particularly important in MetS. Incorporating indicators that better reflect central obesity may therefore further improve the predictive value of the TyG index for adverse outcomes.

To address this limitation, Krakauer et al. introduced the A Body Shape Index (ABSI), which standardizes waist circumference by height and BMI. This index more accurately reflects abdominal fat distribution independent of total body size and has been independently associated with all-cause mortality [16]. Building on this concept, a novel composite indicator, TyG-ABSI, was developed by combining the TyG index with ABSI. This indicator integrates both IR and central obesity into a single measure. Recent studies suggest that TyG-ABSI may outperform traditional TyG-related indices in predicting cardiovascular mortality [17] and has shown promising prognostic value in populations with hyperuricemia [18].

Although TyG-ABSI has demonstrated prognostic value in the general population, its utility in individuals with MetS remains unclear. Given the complex metabolic disturbances often present in MetS and the limitations of existing TyG-related indices in fully capturing these abnormalities, it is essential to evaluate whether TyG-ABSI more effectively reflects metabolic burden and serves as a valuable complement to traditional risk markers. Specifically, assessing its associations with key adverse outcomes, such as the prevalence of CVD (cross-sectional) and cardiovascular and all-cause mortality (longitudinal), is crucial for improving risk identification and advancing precision prevention strategies in the MetS population.

Therefore, this study used data from the nationally representative National Health and Nutrition Examination Survey (NHANES) to explore the associations between TyG-ABSI and CVD, cardiovascular mortality, and all-cause mortality among U.S. adults with MetS. A complex survey-weighted analytical approach was employed to ensure national representativeness. The study aims to clarify the clinical relevance of TyG-ABSI in relation to cardiovascular events in this population and compare it with the traditional TyG index to assess its value in this specific group.

## Materials and methods

### Study design and population

This study used data from the NHANES, a nationally representative program designed to assess the health and nutritional status of the civilian, non-institutionalized U.S. population. NHANES employs a stratified, multistage probability sampling design [19], which helps ensure the representativeness of the findings. All procedures followed the ethical principles outlined in the Declaration of Helsinki and were approved by the Ethics Review Board of the National Center for Health Statistics [20]. Written informed consent was obtained from all participants. Detailed methodology is available on the official NHANES website (https://wwwn.cdc.gov/nchs/nhanes/Default.aspx).

**Fig. 1:**
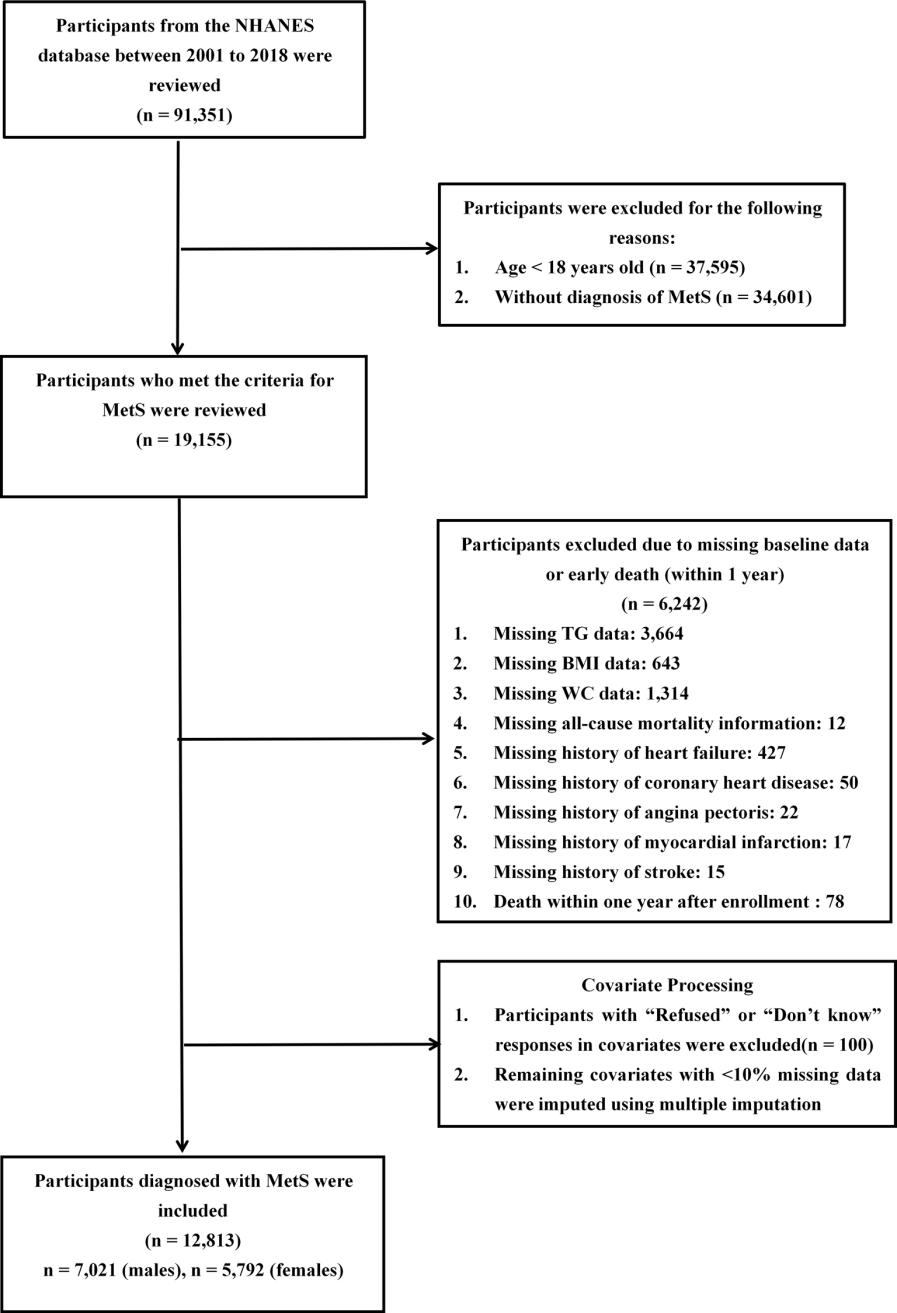
Flow diagram of participants selection. Abbreviation: MetS: metabolic syndrome; TG: triglyceride; BMI: body mass index; WC: waist circumference

Data were obtained from nine continuous NHANES cycles (2001–2018), initially including 91,351 participants. After excluding individuals under 18 years of age (n = 37,595) and those not meeting the criteria for MetS (n = 34,601), 19,155 participants remained. An additional 6,242 individuals were excluded due to missing baseline data or death within one year of enrollment. A further 100 participants were excluded for responding “Refused” or “Don’t know” to key questionnaire items. Covariates with less than 10% missing data were addressed using multiple imputation. The final analytic sample included 12,813 participants with MetS (7,021 males and 5,792 females) (Fig. 1).

### Definition of MetS

According to the criteria established by the National Cholesterol Education Program Adult Treatment Panel III (NCEP ATP III) [21], individuals were defined as having MetS if they met at least three of the following five components: (1) fasting blood glucose (FBG) >100 mg/dL or current treatment for diabetes mellitus [22]; (2) HDL-C < 50 mg/dL in females or < 40 mg/dL in males, or use of medications to increase HDL-C levels [23]; (3) TG >150 mg/dL or current treatment for hypertriglyceridemia [24]; (4) waist circumference (WC) >88 cm in females or >102 cm in males [25]; and (5) systolic blood pressure >130 mmHg and/or diastolic blood pressure >85 mmHg, or current use of antihypertensive medication [26].

### Definition of the TyG index, TyG-ABSI, and related derivatives

The TyG index, calculated as ln [TG × FBG/2], is a well-established and convenient surrogate marker for IR [27, 28]. Several TyG-derived indices have been developed by integrating TyG with anthropometric measures indicative of obesity and body composition. One such derivative is TyG-ABSI, which combines the TyG index with a waist circumference metric standardized by height and BMI [17, 18]. Additional indices include TyG-BMI, TyG-WC, and TyG-WHtR, calculated by multiplying the TyG index by BMI, WC, or WC/height, respectively.

Participants were divided into tertiles based on the distribution of each index, with the lowest tertile (T1) serving as the reference group. T1 represents the lowest, T2 the intermediate, and T3 the highest level.

Formulas for TyG-related indices:

$$ {\text{TyG}} = {\text{ln }}\left[ {{\text{TG}} \times {\text{FBG}}/{2}} \right] $$

$$ {\text{TyG - BMI}} = {\text{TyG}} \times {\text{BMI}} $$

$$ {\text{TyG - WC}} = {\text{TyG}} \times {\text{WC}} $$

$$ {\text{TyG - WHtR}} = {\text{TyG}} \times {\text{WC}}/{\text{height}} $$

$$ {\text{TyG - ABSI}} = {\text{TyG}} \times {\text{WC}}/\left( {{\text{BMI}}^{{{2}/{3}}} \times {\text{height}}^{{{1}/{2}}} } \right) $$

### Covariates of interest

In terms of all demographic and clinical data sourced from the NHANES database, socioeconomic variables encompassed sex (male or female), age, race/ethnicity (Mexican American, other Hispanic, non-Hispanic White, non-Hispanic Black, or other), marital status (single, married, or cohabiting), educational attainment (high school or less, college, or above), and the family poverty-to-income ratio (PIR) (< 1.3, 1.3–3.5, or >3.5).

This study continued to assess behavioral factors and comorbidities. Smoking behavior was classified as non-smoker (< 100 cigarettes in a lifetime) or smoker (≥ 100 cigarettes). Alcohol consumption was defined as non-drinker (< 12 alcoholic beverages per year) or drinker (≥ 12 drinks annually). The presence of cancer and chronic kidney disease (CKD) was determined by self-reported diagnosis (yes or no).

Additional covariates derived from physical examinations and laboratory evaluations were included to account for potential confounding, such as BMI, average daily energy intake (calculated from two 24-h dietary recalls), and a range of biochemical markers, including serum creatinine (Cr), blood urea nitrogen (BUN), total cholesterol (TC), HDL-C, low-density lipoprotein cholesterol (LDL-C), uric acid (UA), alanine aminotransferase (ALT), aspartate aminotransferase (AST), albumin (ALB), and total bilirubin (TBil).

### Outcome definitions

Three primary outcomes were examined: CVD, cardiovascular mortality, and all-cause mortality.

CVD was defined based on self-reported physician diagnoses of at least one of the following: coronary heart disease, angina pectoris, myocardial infarction, heart failure, or stroke, as recorded in the NHANES medical history section.

Mortality outcomes were derived from the NHANES Public-Use Linked Mortality File, with follow-up through December 31, 2019. This database links NHANES participants to the National Death Index using probabilistic matching, overseen by the National Center for Health Statistics. Causes of death were classified according to the International Classification of Diseases, 10th Revision (ICD-10). All-cause mortality was defined as death from any cause (ICD-10 code: 010), while cardiovascular mortality included deaths attributed to heart diseases (ICD-10 codes: 054–068) or cerebrovascular diseases (ICD-10 code: 070).

### Statistical analysis

All statistical analyses followed the analytic guidelines provided by the U.S. Centers for Disease Control and Prevention (CDC) for NHANES (https://wwwn.cdc.gov/nchs/nhanes/tutorials/default.aspx). Given NHANES’s complex, multistage, and stratified probability sampling design, all analyses incorporated sample weights, clustering, and stratification to ensure national representativeness.

Associations between TyG-ABSI and the three main outcomes (CVD, cardiovascular mortality, and all-cause mortality) were evaluated among individuals with MetS. Participants were categorized into tertiles based on baseline TyG-ABSI levels. Complex survey-weighted multivariable models were constructed to estimate these associations. For cardiovascular and all-cause mortality, weighted Cox proportional hazards models were used to calculate hazard ratios (HRs) and 95% confidence intervals (CIs). The proportional hazards assumption was tested using Schoenfeld residuals (Supplementary Table 1).

To address potential competing risks between cardiovascular and non-cardiovascular deaths, Fine and Gray subdistribution hazard models were applied for sensitivity analysis. For the cross-sectional outcome of CVD prevalence, weighted multivariable logistic regression models were used, with results expressed as odds ratios (ORs) and 95% CIs. All models accounted for NHANES’s complex sampling design.

Additional analyses included weighted Kaplan–Meier survival curves, restricted cubic spline (RCS) regression to assess non-linear associations, and subgroup analyses using weighted replication methods to test for effect modification.

Model performance was compared among TyG-ABSI, TyG, and its derived indices (TyG-BMI, TyG-WC, and TyG-WHtR) using receiver operating characteristic (ROC) curves, net reclassification improvement (NRI), integrated discrimination improvement (IDI), and decision curve analysis (DCA).

Covariate selection was informed by prior studies involving TyG-related indices and metabolic risk [14, 29], as well as components of the Framingham Risk Score. Model 1 was unadjusted. Model 2 was adjusted for age, gender, and race. Model 3 (fully adjusted) included marital status, educational level, PIR, smoking, alcohol consumption, cancer history, CKD, BMI (excluded from the TyG-BMI model), total energy intake, and the use of antidiabetic, statin, and antihypertensive medications. It also incorporated biochemical markers: TC, LDL-C, BUN, UA, Cr, ALT, AST, ALB, and TBil.

Systolic blood pressure (SBP) was excluded from the fully adjusted model to avoid overadjustment, as it is already a component of the MetS definition. HDL-C was excluded due to a variance inflation factor (VIF) >5, indicating multicollinearity.

To further evaluate multicollinearity, VIF values were calculated for all covariates [30]. The inclusion of both TC and HDL-C in the same model resulted in VIF >5, suggesting strong collinearity; thus, HDL-C was excluded from all fully adjusted models, retaining only TC. Similarly, in the TyG-BMI model, BMI was excluded due to a VIF >5 to prevent biased estimates.

Subgroup analyses were performed across categories such as age (< 65 vs. ≥ 65 years), gender, marital status, educational level, PIR, smoking, alcohol use, CKD, cancer history, and medication use (antidiabetic, statin, antihypertensive). Interaction terms were tested using likelihood ratio tests.

To assess the robustness of the findings, E-value analysis was conducted to quantify the minimum strength of an unmeasured confounder required to explain away the observed associations [31, 32].

All analyses were performed using SPSS software (version 26.0; IBM Corp., Armonk, NY, USA) and Free Statistics software (version 2.0) [33].

## Results

### Differences in baseline characteristics across TyG-ABSI tertiles

Baseline characteristics of the 12,813 participants were analyzed according to TyG-ABSI tertiles (T1: lowest; T2: middle; T3: highest). As shown in Table 1, higher TyG-ABSI levels were associated with progressively unfavorable demographic, metabolic, and clinical profiles. Participants in T3 were significantly older (57.17 ± 16.12 years vs. 45.18 ± 16.06 years in T1, p < 0.001), more likely to be male (63.76% vs. 41.86%, p < 0.001), had lower educational attainment (below high school: 31.47% vs. 20.16%, p < 0.001), and a higher prevalence of economic disadvantage (PIR < 1.3: 33.39% vs. 29.69%, p < 0.001).

**Table 1:** Demographic characteristics of individuals with MetS stratified by TyG-ABSI tertiles (weighted)

Variables	Total (n = 12,813)	T1 (lowest) (n = 4271)	T2 (middle) (n = 4271)	T3 (highest) (n = 4271)	P-value
Age (years)	51.65 ± 16.95	45.18 ± 16.06	52.59 ± 16.45	57.17 ± 16.12	< 0.001
*Gender (%)*					< 0.001
Male	7021 (54.80)	1788 (41.86)	2510 (58.77)	2723 (63.76)	
Female	5792 (45.20)	2483 (58.14)	1761 (41.23)	1548 (36.24)	
*Race (%)*					< 0.001
Mexican American	2223 (17.35)	586 (13.72)	814 (19.06)	823 (19.27)	
Other Hispanic	995 ( 7.77)	280 (6.56)	354 (8.29)	361 (8.45)	
Non-Hispanic White	6131 (47.85)	1602 (37.51)	2110 (49.4)	2419 (56.64)	
Non-Hispanic Black	2866 (22.37)	1632 (38.21)	787 (18.43)	447 (10.47)	
Other race-including multi-racial	598 ( 4.67)	171 (4)	206 (4.82)	221 (5.17)	
*Marital status (%)*					< 0.001
Single	4750 (37.07)	1808 (42.33)	1463 (34.25)	1479 (34.63)	
Married/living with partner	8063 (62.93)	2463 (57.67)	2808 (65.75)	2792 (65.37)	
*Education level (%)*					< 0.001
Below high school	3336 (26.04)	861 (20.16)	1131 (26.48)	1344 (31.47)	
High school graduate	3234 (25.24)	1095 (25.64)	1072 (25.1)	1067 (24.98)	
Above high school	6243 (48.72)	2315 (54.2)	2068 (48.42)	1860 (43.55)	
*PIR (%)*					< 0.001
< 1.3	4050 (31.61)	1268 (29.69)	1356 (31.75)	1426 (33.39)	
1.3–3.5	5013 (39.12)	1694 (39.66)	1611 (37.72)	1708 (39.99)	
≥ 3.5	3750 (29.27)	1309 (30.65)	1304 (30.53)	1137 (26.62)	
*Smoking status (%)*					< 0.001
Yes	6300 (49.17)	1697 (39.73)	2183 (51.11)	2420 (56.66)	
No	6513 (50.83)	2574 (60.27)	2088 (48.89)	1851 (43.34)	
*Alcohol use (%)*					0.059
Yes	9008 (70.30)	2942 (68.88)	3055 (71.53)	3011 (70.50)	
No	3805 (29.70)	1329 (31.12)	1216 (28.47)	1260 (29.50)	
*Cancer (%)*					< 0.001
Yes	1289 (10.06)	293 (6.86)	429 (10.04)	567 (13.28)	
No	11,524 (89.94)	3978 (93.14)	3842 (89.96)	3704 (86.72)	
*CKD (%)*					< 0.001
Yes	386 ( 3.01)	82 (1.92)	123 (2.88)	181 (4.24)	
No	12,427 (96.99)	4189 (98.08)	4148 (97.12)	4090 (95.76)	
BMI(kg/m^2^)	34.98 ± 6.10	37.17 ± 6.71	34.56 ± 5.54	33.21 ± 5.26	< 0.001
Energy intake, M (Q1, Q3)	1884(1271, 2565)	1878(1254.50, 2560.50)	1908(1263, 2583)	1877(1295, 2545.50)	0.63
HDL-C (mg/dL)	47.74 ± 13.40	52.15 ± 13.37	47.81 ± 12.92	43.27 ± 12.40	< 0.001
LDL-C (mg/dL)	115.93 ± 35.48	116.04 ± 34.97	116.78 ± 35.52	114.97 ± 35.94	0.061
BUN (mg/dL)	13.87 ± 6.13	12.59 ± 5.04	14.04 ± 5.93	14.98 ± 7.02	< 0.001
UA (mg/dL)	5.92 ± 1.44	5.69 ± 1.36	6.03 ± 1.42	6.05 ± 1.50	< 0.001
Cr (mg/dL)	0.92 ± 0.38	0.88 ± 0.34	0.93 ± 0.42	0.95 ± 0.38	< 0.001
ALT, M (Q1, Q3)	23.00 (17.00, 32.00)	21.00 (16.00, 29.00)	24.00 (18.00, 33.00)	24.00 (18.00, 34.00)	< 0.001
AST, M (Q1, Q3)	23.00 (19.00, 28.00)	22.00 (18.00, 27.00)	23.00 (20.00, 28.00)	24.00 (20.00, 30.00)	< 0.001
ALB (g/dL)	4.14 ± 0.35	4.10 ± 0.34	4.16 ± 0.34	4.15 ± 0.37	< 0.001
TBil, M (Q1, Q3)	0.60 (0.50, 0.90)	0.60 (0.50, 0.80)	0.70 (0.50, 0.90)	0.60 (0.50, 0.90)	< 0.001
TG, M (Q1, Q3)	147.00 (100.00, 219.00)	92.00 (71.00, 121.00)	147.00 (114.00, 191.00)	240.00 (178.00, 334.50)	< 0.001
FBG (mg/dl)	107.81 ± 40.92	93.59 ± 15.48	101.14 ± 23.99	128.68 ± 59.38	< 0.001
TC (mg/dL)	196.02 ± 43.14	186.49 ± 37.18	195.58 ± 40.40	205.97 ± 48.78	< 0.001
*Antidiabetic medication use (%)*					< 0.001
No	12,314 (96.11)	4221 (98.83)	4141 (96.96)	3952 (92.53)	
Yes	499 ( 3.89)	50 (1.17)	130 (3.04)	319 (7.47)	
*Statin use (%)*					< 0.001
No	9922 (77.44)	3709 (86.84)	3291 (77.05)	2922 (68.41)	
Yes	2891 (22.56)	562 (13.16)	980 (22.95)	1349 (31.59)	
*Antihypertensive use (%)*					< 0.001
Yes	11,147 (87.00)	3637 (85.16)	3711 (86.89)	3799 (88.95)	
No	1666 (13.00)	634 (14.84)	560 (13.11)	472 (11.05)	
CVD(%)					< 0.001
No	11,173 (87.20)	3944 (92.34)	3740 (87.57)	3489 (81.69)	
Yes	1640 (12.80)	327 (7.66)	531 (12.43)	782 (18.31)	
*All-cause mortality (%)*					< 0.001
No	11,063 (86.34)	3946 (92.39)	3708 (86.82)	3409 (79.82)	
Yes	1750 (13.66)	325 (7.61)	563 (13.18)	862 (20.18)	
*Cardiovascular mortality (%)*					< 0.001
No	12,268 (95.75)	4176 (97.78)	4094 (95.86)	3998 (93.61)	
Yes	545 ( 4.25)	95 (2.22)	177 (4.14)	273 (6.39)	

MetS: metabolic syndrome; TyG-ABSI: triglyceride-glucose and a body shape index; CVD: cardiovascular disease; PIR: family poverty-to-income ratio; BMI: body mass index; TC: total cholesterol; HDL-C: high-density lipoprotein cholesterol; LDL-C: low-density lipoprotein cholesterol; BUN: blood urea nitrogen; UA: uric acid; Cr: creatinine; ALT: alanine aminotransferase; AST: aspartate transaminase; ALB: albumin; TBil: total bilirubin; TG: triglyceride; FBG: fasting blood glucose; CKD: chronic kidney disease; M: median; Q1: 1st quartile; Q3: 3rd quartile

Metabolic parameters also demonstrated a clear gradient across TyG-ABSI tertiles. Compared with T1, individuals in T3 had significantly higher FBG (128.68 ± 59.38 mg/dL vs. 93.59 ± 15.48 mg/dL, p < 0.001), elevated TG [median 240.00 mg/dL (IQR: 178.00–334.50) vs. 92.00 mg/dL (71.00–121.00), p < 0.001], lower HDL-C (43.27 ± 12.40 mg/dL vs. 52.15 ± 13.37 mg/dL, p < 0.001), higher creatinine (0.95 ± 0.38 mg/dL vs. 0.88 ± 0.34 mg/dL, p < 0.001), and elevated uric acid (6.05 ± 1.50 mg/dL vs. 5.69 ± 1.36 mg/dL, p < 0.001), indicating increased metabolic burden.

A similar trend was observed in clinical outcomes. The prevalence of CVD in T3 was 18.31% compared to 7.66% in T1 (2.4-fold increase). All-cause mortality was 20.18% in T3 vs. 7.61% in T1 (2.7-fold increase), and cardiovascular mortality was 6.39% vs. 2.22% (2.9-fold increase), with all differences statistically significant (p < 0.001). Medication usage followed the same pattern: antidiabetic drug use was 7.47% in T3 vs. 1.17% in T1, and statin use was 31.59% vs. 13.16%.

### Associations between TyG-ABSI and the risk of CVD, cardiovascular mortality, and all-cause mortality

Survey-weighted multivariable regression models were used to evaluate associations between TyG-ABSI and adverse outcomes. CVD risk was assessed using logistic regression, while mortality outcomes were examined via Cox proportional hazards models. Three models with increasing levels of covariate adjustment were constructed, with Model 3 representing the fully adjusted model incorporating demographic, socioeconomic, clinical, laboratory, and medication-related variables. As shown in Table 2, TyG-ABSI was significantly and positively associated with the risk of CVD, cardiovascular mortality, and all-cause mortality.

**Table 2:** Complex survey-weighted regression analysis of TyG-ABSI and risks of CVD, cardiovascular mortality, and all-cause mortality

Exposure	Outcome	Model 1		Model 2		Model 3	
		OR(95%CI)	P-value	OR(95%CI)	p-value	OR(95%CI)	p-value
TyG-ABSI	CVD	1.89 (1.73 ~ 2.06)	< 0.001	1.39 (1.25 ~ 1.55)	< 0.001	1.28 (1.15 ~ 1.43)	< 0.001
*TyG-ABSI tertile*							
T1 (lowest)		Reference		Reference		Reference	
T2 (middle)		1.77 (1.48 ~ 2.12)	< 0.001	1.23 (1.00 ~ 1.50)	0.048	1.09 (0.89 ~ 1.35)	0.396
T3 (highest)		3.19 (2.66 ~ 3.82)	< 0.001	1.75 (1.43 ~ 2.14)	< 0.001	1.48 (1.20 ~ 1.83)	< 0.001
P for trend		< 0.001		< 0.001		< 0.001	
		HR(95%CI)	P-value	HR(95%CI)	p-value	HR(95%CI)	p-value
TyG-ABSI	Cardiovascular mortality	1.83 (1.63 ~ 2.06)	< 0.001	1.26 (1.10 ~ 1.47)	0.003	1.25 (1.08 ~ 1.45)	0.003
*TyG-ABSI tertile*							
T1 (lowest)		Reference		Reference		Reference	
T2 (middle)		1.99 (1.46 ~ 2.71)	< 0.001	1.18 (0.87 ~ 1.62)	0.288	1.25 (0.90 ~ 1.73)	0.193
T3 (highest)		3.11 (2.35 ~ 4.12)	< 0.001	1.37 (1.01 ~ 1.85)	0.043	1.40 (1.02 ~ 1.92)	0.036
P for trend		< 0.001		0.037		0.032	
TyG-ABSI	All-cause mortality	1.85 (1.72 ~ 1.98)	< 0.001	1.30 (1.20 ~ 1.42)	< 0.001	1.28 (1.16 ~ 1.40)	< 0.001
*TyG-ABSI tertile*							
T1 (lowest)		Reference		Reference		Reference	
T2 (middle)		1.71 (1.43 ~ 2.05)	< 0.001	1.06 (0.89 ~ 1.27)	0.514	1.08 (0.88 ~ 1.31)	0.478
T3 (highest)		2.92 (2.52 ~ 3.38)	< 0.001	1.37 (1.17 ~ 1.60)	< 0.001	1.34 (1.11 ~ 1.61)	0.002
P for trend		< 0.001		< 0.001		< 0.001	

Model 1: No adjustment; Model 2: Adjusted for age, gender, and race; Model 3: Additionally adjusted for marital status, education, PIR, smoking, alcohol use, cancer, CKD, BMI, total energy intake, TC, LDL-C, BUN, UA, Cr, ALT, AST, ALB, TBil, antidiabetic medication use, statin use, antihypertensive use

In continuous analyses, each one-unit increase in TyG-ABSI was associated with a 28% higher risk of CVD (OR = 1.28; 95% CI: 1.15–1.43; p < 0.001), a 25% higher risk of cardiovascular mortality (HR = 1.25; 95% CI: 1.08–1.45; p = 0.003), and a 28% higher risk of all-cause mortality (HR = 1.28; 95% CI: 1.16–1.40; p < 0.001).

In tertile-based analyses, compared to individuals in T1, those in T3 had a 48% higher risk of CVD (OR = 1.48; 95% CI: 1.20–1.83; p < 0.001), a 40% higher risk of cardiovascular mortality (HR = 1.40; 95% CI: 1.02–1.92; p = 0.036), and a 34% higher risk of all-cause mortality (HR = 1.34; 95% CI: 1.11–1.61; p = 0.002). All trend p-values were statistically significant (CVD and all-cause mortality: p for trend < 0.001; cardiovascular mortality: p for trend = 0.032), indicating robust dose–response relationships.

Sensitivity analysis using the Fine–Gray subdistribution hazard model confirmed a significant positive association between TyG-ABSI and cardiovascular mortality after full adjustment (Model 3). These findings were consistent with those from the primary Cox models, suggesting that competing risks did not materially alter the observed associations (Supplementary Table 2).

As shown in Supplementary Table 3, among all TyG-related indices (TyG, TyG-WC, TyG-WHtR, and TyG-BMI), only TyG-ABSI consistently demonstrated significant associations with CVD, cardiovascular mortality, and all-cause mortality in both continuous and categorical analyses.

### Comprehensive evaluation of the predictive performance of TyG-ABSI

#### ROC analysis of TyG-ABSI and related indices

**Fig. 2:**
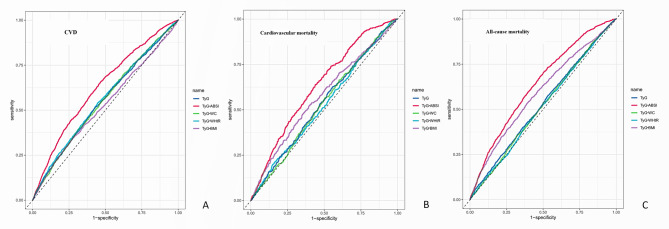
ROC curves highlighting the superior predictive performance of TyG-ABSI for the risk of CVD (**A**), cardiovascular mortality (**B**), and all-cause mortality (**C**). Abbreviation: CVD: cardiovascular disease

Based on the ROC curves (Fig. 2A–C) and corresponding AUC values (Supplementary Table 4), TyG-ABSI consistently exhibited the strongest diagnostic efficacy among all TyG-related indices across the three clinical outcomes.

For CVD (Fig. 2A), TyG-ABSI showed the highest discriminative ability, with an AUC of 62.22% (95% CI: 60.79%–63.66%), outperforming TyG (55.28%), TyG-WC (55.16%), TyG-WHtR (55.43%), and TyG-BMI (52.54%). The optimal cutoff value for TyG-ABSI was determined to be 7.47, indicating that a TyG-ABSI value greater than 7.47 may be useful for predicting the occurrence of CVD.

In predicting cardiovascular mortality (Fig. 2B), TyG-ABSI again demonstrated the best performance, with an AUC of 63.46% (95% CI: 61.21%–65.71%), superior to TyG-BMI (58.28%), TyG (53.55%), TyG-WC (52.07%), and TyG-WHtR (51.82%). The optimal cutoff value for TyG-ABSI was determined to be 7.54, indicating that a TyG-ABSI value greater than 7.54 may be useful for predicting the occurrence of cardiovascular mortality.

For all-cause mortality (Fig. 2C), TyG-ABSI achieved the highest AUC of 64.12% (95% CI: 62.77%–65.47%), outperforming TyG-BMI (59.03%) and significantly exceeding the performance of the other indices, all of which had AUCs below 54%. The optimal cutoff value for TyG-ABSI was determined to be 7.39, indicating that a TyG-ABSI value greater than 7.39 may be useful for predicting the occurrence of all-cause mortality.

#### Incremental predictive value of TyG-ABSI and related indices

To evaluate the incremental predictive value of TyG-ABSI and related indices beyond traditional risk factors, improvements in risk reclassification and model discrimination were assessed using NRI and IDI metrics (Table 3). The baseline model was based on Framingham risk factors (Model 3).

**Table 3:** Incremental predictive value of TyG-ABSI and related indices

	C-statistic (95%CI)	P value	Continunous NRI (95%CI)	P value	IDI (95%CI)	P value
*CVD*						
Basic model	0.8336 (0.8241 ~ 0.8432)		Ref		Ref	
Basic model + TyG-ABSI	0.8346 (0.8251 ~ 0.8441)	0.010	0.123 (0.071–0.175)	< 0.001	8e-04 (–1e-04–0.002)	0.092
Basic model + TyG	0.8345 (0.8250 ~ 0.8440)	0.037	0.097 (0.045–0.149)	< 0.001	9e-04 (–1e-04–0.002)	0.082
Basic model + TyG-WC	0.8344 (0.8249 ~ 0.8439)	0.046	0.105 (0.053–0.157)	< 0.001	5e-04 (–3e-04–0.001)	0.240
Basic model + TyG-WHtR	0.8345 (0.8251 ~ 0.8441)	0.023	0.115 (0.064–0.167)	< 0.001	0.001 (0.000–0.003)	0.064
Basic model + TyG-BMI	0.8344 (0.8250 ~ 0.8440)	0.035	0.084 (0.033–0.136)	0.001	9e-04 (–1e-04–0.002)	0.074
*Cardiovascular mortality*						
Basic model	0.9166 (0.9001 ~ 0.9330)		Ref		Ref	
Basic model + TyG-ABSI	0.9182 (0.9019 ~ 0.9345)	0.033	0.105 (0.003–0.154)	0.044	0.004(0 ~ 0.01)	0.080
Basic model + TyG	0.9168 (0.9004 ~ 0.9332)	0.949	0.119 (–0.063–0.175)	0.100	0.002(0 ~ 0.06)	0.164
Basic model + TyG-WC	0.9180 (0.9018 ~ 0.9343)	0.026	0.100 (–0.003–0.164)	0.060	0.003(0 ~ 0.09)	0.076
Basic model + TyG-WHtR	0.9181 (0.9017 ~ 0.9344)	0.091	0.830 (–0.002–0.141)	0.064	0.004(0 ~ 0.01)	0.078
Basic model + TyG-BMI	0.9169 (0.9006 ~ 0.9333)	0.749	0.111 (–0.041–0.173)	0.080	0.002(0 ~ 0.07)	0.076
*All-cause mortality*						
Basic model	0.8936 (0.8809 ~ 0.9063)		Ref		Ref	
Basic model + TyG-ABSI	0.8961 (0.8836 ~ 0.9086)	0.013	0.096 (0.046–0.136)	0.004	0.006(0.002 ~ 0.01)	< 0.001
Basic model + TyG	0.8939 (0.8813 ~ 0.9066)	0.613	0.077 (–0.009–0.120)	0.068	0.002(0 ~ 0.05)	0.104
Basic model + TyG-WC	0.8956 (0.8830 ~ 0.9081)	0.047	0.093 (0.043–0.139)	0.004	0.006(0.002 ~ 0.01)	< 0.001
Basic model + TyG-WHtR	0.8960 (0.8836 ~ 0.9087)	0.013	0.092 (0.047–0.132)	0.004	0.006(0.002 ~ 0.01)	< 0.001
Basic model + TyG-BMI	0.8941 (0.8554 ~ 0.8736)	0.403	0.076 (–0.002–0.116)	0.052	0.002(0 ~ 0.05)	0.032

Basic model adjustment included: age, gender, race, marital status, education, PIR, smoking, alcohol use, cancer, CKD, BMI, energy intake, antidiabetic, statin, antihypertensive, TC, LDL-C, BUN, UA, Cr, ALT, AST, ALB, TBil

For CVD, adding TyG-ABSI significantly improved risk reclassification (NRI = 0.123, p < 0.001), outperforming all other TyG-related indices. Similarly, for cardiovascular mortality, TyG-ABSI provided the greatest improvement (NRI = 0.105, p = 0.044). In predicting all-cause mortality, TyG-ABSI again outperformed the other indices (NRI = 0.096, p = 0.004).

For IDI, no significant improvement was observed for CVD or cardiovascular mortality (all p >0.05). However, for all-cause mortality, TyG-ABSI, TyG-WC, and TyG-WHtR showed comparable improvements in discrimination (IDI = 0.006, p < 0.001), indicating added predictive value.

#### DCA of TyG-ABSI and Related Indices

**Fig. 3:**
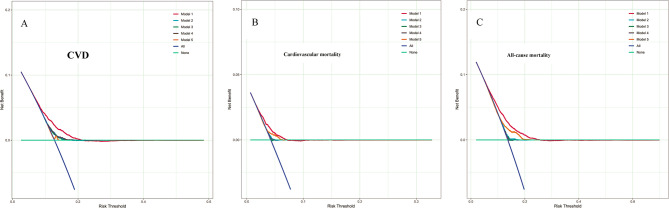
Decision curve analysis comparing TyG-ABSI with other TyG-based indices in predicting CVD, cardiovascular mortality, and all-cause mortality: assessment of net benefit. Model 1: TyG-ABSI; Model 2: TyG; Model 3: TyG-WC; Model 4: TyG-WHtR; Model 5: TyG-BMI; None: No intervention strategy; All: Assume all individuals are treated. **A**: Prediction of CVD; **B**: Prediction of cardiovascular mortality; **C**: Prediction of all-cause mortality

As shown in the decision curve analysis (Fig. 3), TyG-ABSI consistently demonstrated superior net clinical benefit across multiple risk thresholds compared to other TyG-related indices (such as TyG, TyG-WC, TyG-WHtR, and TyG-BMI). Overall, TyG-ABSI provided the most favorable and consistent net benefit profile among the evaluated indices.

### Survival analysis by TyG-ABSI and other indices

**Fig. 4:**
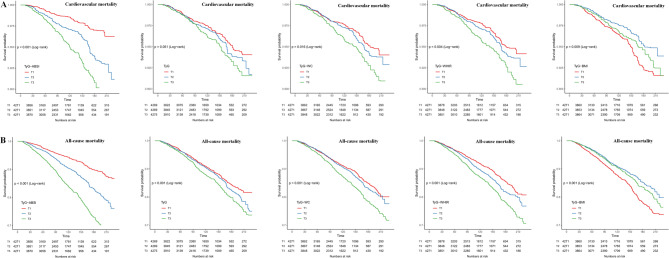
Complex survey-weighted Kaplan–Meier survival curves for TyG-related indices by tertiles: cardiovascular mortality (**A**) and all-cause mortality (**B**). Kaplan–Meier survival analysis comparing the cumulative survival probability across tertiles of TyG-ABSI, TyG, TyG-WC, TyG-WHtR, and TyG-BMI

Figure 4 presents complex survey-weighted Kaplan–Meier survival curves across tertiles of TyG-ABSI and other TyG-related indices (TyG, TyG-WC, TyG-WHtR, and TyG-BMI) for cardiovascular mortality (Panel A) and all-cause mortality (Panel B). Among these, TyG-ABSI showed the most distinct and consistent separation of survival curves across tertiles, with the highest tertile (T3) exhibiting the lowest survival probability and the lowest tertile (T1) maintaining the most favorable prognosis throughout follow-up. In contrast, the survival curves for TyG, TyG-WC, and TyG-WHtR showed limited separation with substantial overlap. Notably, TyG-BMI displayed an inverse trend, where individuals in T1 had lower survival than those in T3. Overall, TyG-ABSI demonstrated clearer risk stratification across tertiles, suggesting its potential utility in identifying long-term mortality risk.

### Tyg-ABSI-based RCS analysis for CVD, cardiovascular mortality, and all-cause mortality

**Fig. 5:**
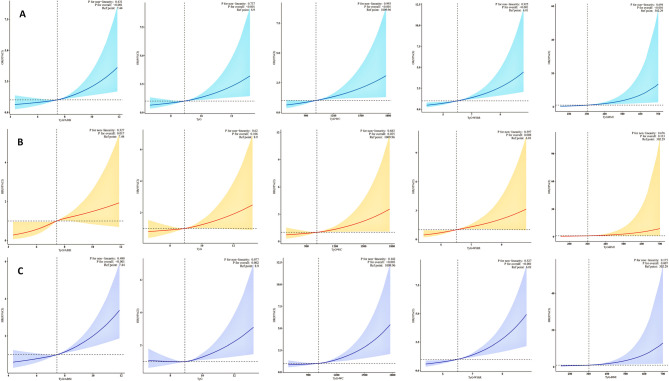
Dose–response associations between TyG-derived indices and adverse outcomes based on complex survey-weighted RCS models. RCS curves illustrate the adjusted associations of TyG-ABSI, TyG-WHtR, TyG-BMI, TyG-WC, and TyG with three adverse outcomes: **A** CVD, **B** cardiovascular mortality, and **C** all-cause mortality. Models were adjusted for gender, age, race, marital status, education, PIR, smoking, alcohol use, cancer, CKD, BMI (excluded in the TyG-BMI analysis), total energy intake, and biochemical markers including TC, LDL-C, BUN, UA, Cr, ALT, AST, ALB, and TBil, as well as use of antidiabetic, statin, and antihypertensive medications. Reference point: Median. RCS: Restricted cubic spline. Reference: Ref

Statistical models were selected based on outcome type, and restricted cubic spline (RCS) functions were incorporated to examine the dose–response relationships of TyG-related indices. As shown in Fig. 5 (A: CVD; B: cardiovascular mortality; C: all-cause mortality), complex survey-weighted logistic regression was applied to the cross-sectional outcome (CVD), while complex survey-weighted Cox proportional hazards models were used for longitudinal outcomes (cardiovascular and all-cause mortality). RCS functions were then added to each model to assess potential nonlinearity and visualize dose–response patterns across the range of continuous exposures.

Across all three outcomes, TyG-ABSI demonstrated a stable and approximately linear association with increased risk. Tests for non-linearity were not statistically significant (p for non-linearity >0.05), while the overall associations remained significant (p for overall < 0.05), indicating strong linear predictive ability and good model stability when TyG-ABSI was treated as a continuous variable (Fig. 5A–C).

Other TyG-related indices, including TyG, TyG-WC, TyG-WHtR, and TyG-BMI, also exhibited similar linear trends (p for non-linearity >0.05) and consistent dose–response patterns across models. However, in RCS models for cardiovascular mortality, TyG and TyG-BMI did not show statistically significant overall associations (p for overall >0.05), suggesting relatively limited risk stratification value for this specific outcome.

### E-value analysis for robustness of the associations between TyG-ABSI and adverse outcomes

E-values were calculated to evaluate the potential influence of unmeasured confounding on the associations between TyG-ABSI and three major outcomes, CVD, cardiovascular mortality, and all-cause mortality, using both continuous and tertile-based multivariable regression models. E-values ranged from 1.81 to 2.32.

For CVD, the E-value was 1.88 in the continuous model and 2.32 for the T3 versus T1 comparison. For cardiovascular mortality, the corresponding values were 1.81 and 2.15, and for all-cause mortality, 1.88 and 2.01, respectively. All E-values were close to or exceeded the commonly accepted robustness threshold of 1.82 [32], supporting the credibility and robustness of the observed associations.

### Subgroup analyses of TyG-ABSI in relation to CVD, cardiovascular mortality, and all-cause mortality

**Fig. 6:**
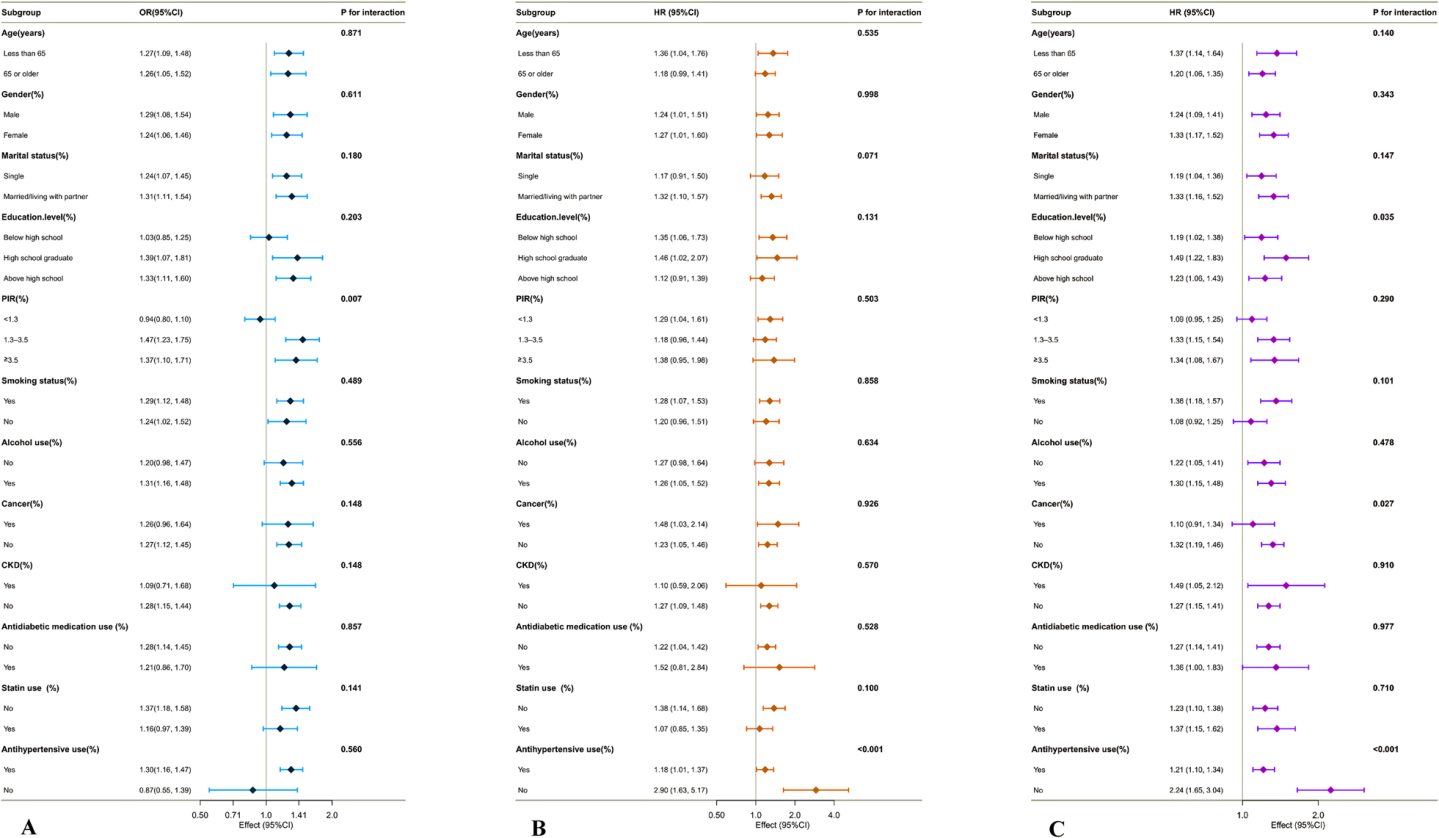
Subgroup analyses of the associations between TyG-ABSI and adverse outcomes based on weighted regression with complex sampling. **A**–**C** illustrate the subgroup analyses of TyG-ABSI in relation to **A** CVD, **B** cardiovascular mortality, and **C** all-cause mortality. Most subgroups demonstrated consistent associations without significant interaction effects. All models were adjusted for age, gender, race, marital status, education, PIR, smoking, alcohol use, cancer, CKD, BMI, total energy intake, TC, LDL-C, BUN, UA, Cr, ALT, AST, ALB, TBil, and use of antidiabetic, statin, and antihypertensive Medications

As shown in Fig. 6, the associations between TyG-ABSI and adverse outcomes remained statistically significant across most subgroups, with no evidence of significant interactions (p for interaction >0.05), indicating generally consistent predictive performance across population strata. All analyses were conducted using a complex survey-weighted design, with logistic regression models applied for CVD (cross-sectional outcome) and Cox proportional hazards models for mortality outcomes (longitudinal outcomes).

In the CVD analysis (Fig. 6A), a significant interaction was observed only in the subgroup stratified by PIR (p for interaction = 0.007). Among participants with PIR < 1.3, the association between TyG-ABSI and CVD was not statistically significant (OR = 0.94; 95% CI: 0.80–1.10). In contrast, significant associations were found among those with PIR between 1.3 and 3.5 (OR = 1.47; 95% CI: 1.23–1.75) and PIR ≥ 3.5 (OR = 1.37; 95% CI: 1.10–1.71), suggesting stronger associations in middle- and high-income groups.

In the analysis of cardiovascular mortality (Fig. 6B), a significant interaction was identified in the subgroup defined by antihypertensive medication use (p for interaction < 0.001). Among individuals not taking antihypertensive medications, TyG-ABSI was significantly associated with increased cardiovascular mortality (HR = 2.90; 95% CI: 1.63–5.17), whereas the association was attenuated among those receiving treatment (HR = 1.18; 95% CI: 1.01–1.37).

In the all-cause mortality analysis (Fig. 6C), three significant interactions were observed. First, in the education subgroup (p for interaction = 0.035), the hazard ratios were 1.19 (95% CI: 1.02–1.38) for individuals with less than a high school education, 1.49 (95% CI: 1.22–1.83) for high school graduates, and 1.23 (95% CI: 1.06–1.43) for those with education beyond high school. Second, in the cancer history subgroup (p = 0.027), the association was stronger among individuals without cancer (HR = 1.32; 95% CI: 1.19–1.46), while it was not statistically significant in those with a history of cancer (HR = 1.10; 95% CI: 0.91–1.34). Third, a notable interaction was again observed for antihypertensive medication use (p < 0.001), with HRs of 2.24 (95% CI: 1.65–3.04) in non-users and 1.21 (95% CI: 1.10–1.34) in users.

## Discussion

This study is among the first to conduct a systematic comparison between TyG-ABSI and conventional TyG-related indices (TyG, TyG-WC, TyG-WHtR, and TyG-BMI) in predicting CVD and mortality, using nationally representative data from the NHANES cohort (n = 12,813). The results showed that elevated TyG-ABSI levels were significantly linked to higher risks of CVD, cardiovascular mortality, and all-cause mortality. These associations remained robust after adjusting for a comprehensive set of demographic, socioeconomic, clinical, laboratory, and medication-related variables. In continuous analyses, each one-unit increase in TyG-ABSI was associated with a 28% higher risk of CVD, a 25% increase in cardiovascular mortality, and a 28% rise in all-cause mortality. These associations demonstrated a consistent dose–response relationship across tertile-based stratifications. Compared with other TyG-related indices, TyG-ABSI exhibited superior predictive value across ROC curves, NRI, and DCA, particularly within low-to-moderate risk groups. Additional analyses, including competing risk models, E-value estimation, and RCS modeling, further reinforced the robustness and linearity of these associations. Subgroup analyses generally revealed consistent patterns across different population strata, although significant interactions were observed for poverty-income ratio, antihypertensive medication use, education attainment, and cancer history in specific outcomes.

Although numerous studies have examined the association between the TyG index and the risk of CVD and mortality, most have examined TyG in isolation, without incorporating anthropometric parameters [34–38]. This omission may compromise predictive accuracy. For instance, one study reported that TyG failed to identify at-risk individuals aged 20–65 years for cardiovascular or all-cause mortality [34], while another reported no association between TyG and mortality among those aged 65 or older in the general population [39]. Consequently, recent research has increasingly focused on combining TyG with obesity-related measures, such as BMI, WC, and WHtR, to develop composite indices, particularly in studies assessing risks of CVD and mortality [40–42]. However, cross-sectional evaluations of these composite indices have shown varying predictive capacities across different population groups. In some cases, multiple TyG-derived metrics emerged as concurrent risk predictors [40, 41], reflecting considerable population-level heterogeneity. Although one prior study explored the relationship between TyG-related indices and mortality in individuals with MetS [43], it lacked methodological rigor, as it did not apply complex survey weighting or assess cardiovascular outcomes systematically. Moreover, it failed to evaluate incremental predictive value, utilize competing risk models, estimate E-values, conduct decision-curve analysis, or report AUC metrics. Against this backdrop, the present study leverages nationally representative NHANES data to comprehensively examine the association of the newly developed TyG-ABSI index with CVD and mortality risk, while comparing its predictive performance with other established TyG-related indices. Given its recent development, the utility of TyG-ABSI in populations with MetS has remained unclear. Our findings address this gap and underscore its superior predictive capability.

This study indicates that elevated TyG‑ABSI may be linked to a higher risk of cardiovascular events and mortality among individuals with MetS, potentially through multiple interconnected metabolic and vascular pathological mechanisms. In the context of obesity and IR, increased levels of insulin and aldosterone can activate endothelial sodium channels via mineralocorticoid receptors, resulting in excessive sodium influx and stiffening of the cortical actin cytoskeleton. This process contributes to cardiovascular fibrosis and structural remodeling. Simultaneously, reduced endothelial nitric oxide synthase (eNOS) activity diminishes nitric oxide (NO) production and availability, leading to vascular stiffening and impaired vasodilation [44, 45]. As a surrogate marker of IR, the TyG index has been strongly associated with impaired mitochondrial oxidative phosphorylation, decreased ATP production, and excessive accumulation of reactive oxygen species (ROS) [46–49]. These alterations not only worsen defects in insulin signaling but also promote endothelial dysfunction, chronic vascular inflammation, and myocardial energy imbalance, all of which play a critical role in the pathogenesis of atherosclerosis and heart failure [50]. Central obesity, as captured by ABSI, may further exacerbate these adverse effects. Excess visceral and ectopic fat deposits release free fatty acids and pro-inflammatory cytokines, inhibit mitochondrial respiratory chain function, disrupt mitochondrial fusion–fission dynamics, and suppress the expression of PGC‑1α [51, 52]. These processes accelerate lipid accumulation in the vasculature, enhance foam cell formation, and deplete myocardial energy stores. When central obesity coexists with IR, their combined effect on metabolic dysregulation, mitochondrial and endothelial dysfunction, and systemic inflammation may substantially accelerate cardiovascular deterioration and elevate the risk of major adverse events and mortality. In summary, TyG‑ABSI may contribute to structural and functional damage of the vasculature and myocardium through a mechanistic cascade involving IR, mitochondrial dysfunction, endothelial impairment, and central obesity. This proposed pathway offers a potential biological explanation for the observed association between TyG‑ABSI and adverse clinical outcomes, highlighting the need for validation in future prospective and mechanistic studies.

The overall analyses demonstrated strong and consistent associations between TyG‑ABSI and adverse cardiovascular outcomes and mortality, highlighting its potential utility across diverse populations. Previous studies have reported reliable predictive performance of the TyG index in both the Chinese CHARLS cohort and US/UK populations [53–56], supporting its cross-population generalizability. Since TyG‑ABSI incorporates the TyG index as a component, it is reasonable to infer that it may share similar extrapolative properties. However, current evidence on TyG‑ABSI remains limited, primarily drawn from US-based studies focused on uric acid levels and general population cohorts, as well as data from the Chinese CHARLS cohort [17, 18, 57]. The latter’s design, restricted to individuals aged ≥ 45 years and characterized by distinct covariate structures, limits direct comparability with our survey-weighted dataset, which was based on a more comprehensive baseline model. These limitations underscore the need for further validation of TyG‑ABSI across broader demographic and clinical contexts.

Beyond general applicability, our subgroup analyses identified several notable effect modifiers. For CVD outcomes, a significant interaction was observed with the poverty-income ratio (PIR), with associations primarily evident among individuals in middle- and high-income groups (PIR ≥ 1.3). This pattern may reflect socioeconomic influences on metabolic risk expression, whereby variations in diet, fat distribution, lifestyle behaviors, and access to healthcare may enhance the sensitivity of TyG‑ABSI in these populations. In analyses of cardiovascular and all-cause mortality, the use of antihypertensive medications emerged as a consistent effect modifier. Stronger associations were noted among individuals not receiving antihypertensive therapy, suggesting that blood pressure control may attenuate, but not fully negate, the vascular risks linked to metabolic and central adiposity-related pathways. This attenuation could be mediated by improved arterial compliance and endothelial function, both downstream effects of reduced IR and central obesity. Regarding all‑cause mortality (all‑cause mortality), education level also appeared to influence the magnitude of association, with the strongest effects observed among individuals with a high school education. This finding may relate to variations in health literacy, healthcare access, lifestyle practices, and occupational exposures, identifying a potential target group for preventive strategies. In contrast, the weak and non‑significant association between TyG‑ABSI and all‑cause mortality among participants with a history of cancer may reflect the dominant contribution of cancer‑related morbidity and mortality, which may overshadow the relative influence of metabolic factors in this subgroup. Collectively, these results suggest that while TyG‑ABSI shows promise as a generalizable risk marker, the strength of its associations may vary across demographic, socioeconomic, and clinical strata. Future research should aim to replicate these interactions in larger and more diverse populations beyond the US and China, to better establish the global applicability of TyG‑ABSI and refine its role in personalized cardiovascular risk assessment.

A major strength of this study lies in its use of a large, nationally representative NHANES cohort and the application of complex survey-weighted analyses, which enhance the generalizability and statistical robustness of the findings in U.S. adults with MetS. The study comprehensively evaluated a range of anthropometric and metabolic indices and, for the first time, systematically compared TyG‑ABSI with the original TyG index and its derived measures (TyG‑BMI, TyG‑WC, and TyG‑WHtR). Methodologically, the study employed a rigorous and multidimensional analytical approach, including multivariable regression models, stratified subgroup analyses, NRI, DCA, RCS modeling, and E‑value estimation, collectively strengthening the reliability and clinical relevance of the findings.

The limitations of this study are as follows: First, the cardiovascular disease outcome analysis used a cross-sectional design, which limits causal inference and requires cautious interpretation of temporal relationships. Second, the NHANES sample is limited to the U.S. population, and differences in ethnicity, lifestyle, and healthcare access may affect the generalizability and predictive accuracy of the results in other populations. Finally, this study did not explore the interaction effects between the components of TyG and ABSI. Although this was not the primary focus of the current research, understanding these interactions is important for a deeper insight into their biological relationships.

Overall, TyG‑ABSI demonstrated a stronger association with CVD and superior predictive value for both all-cause and cardiovascular mortality compared to traditional indices. Combined with multidimensional assessments and rigorous statistical methods, it shows promising clinical utility for risk stratification. Future prospective, multicenter studies incorporating mediation analysis will be crucial to confirm its mechanisms and validate its applicability across diverse populations, ultimately improving cardiovascular risk evaluation and personalized prevention strategies. Primary healthcare institutions can utilize annual physical examination data and employ Excel-based formula automation to calculate TyG-ABSI. For patients whose TyG-ABSI exceeds the critical threshold, intensified intervention for metabolic syndrome can be initiated.

## Conclusion

In this nationally representative cohort of U.S. adults with MetS from the NHANES database, TyG‑ABSI was independently associated with higher risks of CVD, cardiovascular mortality, and all‑cause mortality. It also outperformed TyG, TyG‑WC, TyG‑BMI, and TyG‑WHtR in predicting both cardiovascular and all-cause mortality. Dose–response analyses revealed an approximately linear relationship between TyG‑ABSI and mortality risk. Our study highlights the value of TyG-ABSI in assessing cardiovascular disease and mortality risk in populations with MetS, providing new evidence for medical practice and public health interventions.

## Supplementary Information

Below is the link to the electronic supplementary material.

Supplementary Material 1

Supplementary Material 2

Supplementary Material 3

Supplementary Material 4

## Data Availability

The datasets produced and analyzed in this research are accessible in the NHANES database (https://wwwn.cdc.gov/nchs/nhanes/default.aspx).
