# Supplementary material for: Evaluative performance of TyG-ABSI versus traditional indices in relation to cardiovascular disease and mortality: evidence from the U.S. NHANES
Source: Cardiovasc Diabetol. 2025 Aug 21;24:344. doi: 10.1186/s12933-025-02902-6 (PMC12372269; doi:10.1186/s12933-025-02902-6)
Supplement: Supplementary file 1 — Supplementary Material 1 [file 12933_2025_2902_MOESM1_ESM.docx]

### ****Proportional hazards assumption test based on Schoenfeld residuals for TyG-related indices with cardiovascular mortality****

| **Variable** | **Chi-square (χ²)** | **df** | **p-value** |
| --- | --- | --- | --- |
| TyG | 0.007 | 1 | 0.934 |
| TyG-ABSI | 1.715 | 1 | 0.190 |
| TyG-WC | 0.254 | 1 | 0.614 |
| TyG-WHtR | 0.798 | 1 | 0.372 |
| TyG-BMI | 0.095 | 1 | 0.758 |
| **Global test** | **5.778** | **5** | **0.328** |

**Interpretation**: All p-values were greater than 0.05, indicating that the proportional hazards assumption was not violated for any of the TyG-related indices or in the global test.

### ****Proportional hazards assumption test based on Schoenfeld residuals for TyG-related indices with all-cause mortality****

| **Variable** | **Chi-square (χ²)** | **df** | **p-value** |
| --- | --- | --- | --- |
| TyG | 0.788 | 1 | 0.375 |
| TyG-ABSI | 0.782 | 1 | 0.377 |
| TyG-WC | 0.534 | 1 | 0.465 |
| TyG-WHtR | 0.244 | 1 | 0.621 |
| TyG-BMI | 3.335 | 1 | 0.068 |
| **Global test** | **10.383** | **5** | **0.065** |

**Interpretation**: All p-values were greater than 0.05, indicating that the proportional hazards assumption was not violated for any of the TyG-related indices or in the global test.
