# Supplementary material for: Evaluative performance of TyG-ABSI versus traditional indices in relation to cardiovascular disease and mortality: evidence from the U.S. NHANES
Source: Cardiovasc Diabetol. 2025 Aug 21;24:344. doi: 10.1186/s12933-025-02902-6 (PMC12372269; doi:10.1186/s12933-025-02902-6)
Supplement: Supplementary file 2 — Supplementary Material 2 [file 12933_2025_2902_MOESM2_ESM.docx]

Association Between TyG-ABSI and Cardiovascular Mortality Based on Fine–Gray Subdistribution Hazard Model

| **Variable** | **Total Sample Size** | **Events (n, %)** | **Crude HR (95% CI)** | **Crude P-value** | **Adjusted HR (95% CI)** | **Adjusted P-value** |
| --- | --- | --- | --- | --- | --- | --- |
| TyG-ABSI | 12,813 | 545 (4.3%) | 1.72 (1.57–1.88) | < 0.001 | 1.18 (1.04–1.35) | 0.013 |

Adjusted for gender, age, race, marital status, education, PIR, smoking, alcohol, cancer, CKD, BMI, energy intake, TC, LDL-C, BUN, UA, Cr, ALT, AST, ALB, TBil, and medication use (antidiabetic, statin, antihypertensive).
