# Supplementary material for: Evaluative performance of TyG-ABSI versus traditional indices in relation to cardiovascular disease and mortality: evidence from the U.S. NHANES
Source: Cardiovasc Diabetol. 2025 Aug 21;24:344. doi: 10.1186/s12933-025-02902-6 (PMC12372269; doi:10.1186/s12933-025-02902-6)
Supplement: Supplementary file 3 — Supplementary Material 3 [file 12933_2025_2902_MOESM3_ESM.docx]

| Complex survey-weighted multivariable regression analysis between TyG-related indices and the risk of CVD | | | | | | |
| --- | --- | --- | --- | --- | --- | --- |
| Exposure | Model 1 | | Model 2 | | Model 3 | |
|  | OR(95%CI) | P-value | OR(95%CI) | P-value | OR(95%CI) | P-value |
| TyG |  |  |  |  |  |  |
| TyG | 1.41 (1.27~1.56) | <0.001 | 1.37 (1.23~1.54) | <0.001 | 1.28 (1.13~1.44) | <0.001 |
| TyG tertile |  |  |  |  |  |  |
| T1 (lowest) | Reference |  | Reference |  | Reference |  |
| T2 (middle) | 1.36 (1.12~1.65) | 0.002 | 1.20 (0.98~1.48) | 0.081 | 1.18 (0.95~1.48) | 0.141 |
| T3 (highest) | 1.72 (1.41~2.10) | <0.001 | 1.56 (1.27~1.91) | <0.001 | 1.42 (1.15~1.75) | 0.001 |
| P for trend |  | <0.001 |  | <0.001 |  | 0.001 |
| TyG-WC |  |  |  |  |  |  |
| TyG-WC | 1 (1~1) | <0.001 | 1 (1~1) | <0.001 | 1 (1~1) | <0.001 |
| TyG-WC tertile |  |  |  |  |  |  |
| T1 (lowest) | Reference |  | Reference |  | Reference |  |
| T2 (middle) | 1.23 (1.01~1.51) | 0.044 | 1.16 (0.94~1.44) | 0.173 | 1.11 (0.88~1.40) | 0.382 |
| T3 (highest) | 1.64 (1.33~2.01) | <0.001 | 1.79 (1.45~2.21) | <0.001 | 1.42 (1.09~1.84) | 0.009 |
| P for trend |  | <0.001 |  | <0.001 |  | 0.01 |
| TyG-WHtR |  |  |  |  |  |  |
| TyG-WHtR | 1.38 (1.27~1.49) | <0.001 | 1.48 (1.35~1.63) | <0.001 | 1.39 (1.21~1.60) | <0.001 |
| TyG-WHtR tertile |  |  |  |  |  |  |
| T1 (lowest) | Reference |  | Reference |  | Reference |  |
| T2 (middle) | 1.37 (1.13~1.66) | 0.001 | 1.33 (1.10~1.61) | 0.004 | 1.24 (1.00~1.54) | 0.053 |
| T3 (highest) | 1.81 (1.49~2.20) | <0.001 | 1.95 (1.57~2.43) | <0.001 | 1.53 (1.17~2.00) | 0.002 |
| P for trend |  | <0.001 |  | <0.001 |  | 0.002 |
| TyG-BMI |  |  |  |  |  |  |
| TyG-BMI | 1 (1~1) | 0.882 | 1 (1~1.01) | <0.001 | 1.01 (1~1.01) | <0.001 |
| TyG-BMI tertile |  |  |  |  |  |  |
| T1 (lowest) | Reference |  | Reference |  | Reference |  |
| T2 (middle) | 0.84 (0.71~1.00) | 0.057 | 1.19 (0.99~1.44) | 0.067 | 1.16 (0.93~1.43) | 0.18 |
| T3 (highest) | 0.95 (0.79~1.14) | 0.589 | 1.72 (1.40~2.12) | <0.001 | 1.48 (1.08~2.02) | 0.014 |
| P for trend |  | 0.585 |  | <0.001 |  | 0.017 |

Model 1: Non-adjusted; Model 2: Adjusted for age, gender, and race; Model 3: Additionally adjusted for marital status, education, PIR, smoking, alcohol use, cancer, CKD, BMI (excluded when analyzing TyG-BMI), total energy intake, TC, LDL-C, BUN, UA, Cr, ALT, AST, ALB, TBil, antidiabetic medication use, statin use, and antihypertensive use.

| Complex survey-weighted multivariable regression analysis between TyG-related indices and the risk of cardiovascular mortality | | | | | | |
| --- | --- | --- | --- | --- | --- | --- |
| Exposure | Model 1 | | Model 2 | | Model 3 | |
|  | HR(95%CI) | P-value | HR(95%CI) | P-value | HR(95%CI) | P-value |
| TyG |  |  |  |  |  |  |
| TyG | 1.23 (1.07~1.42) | 0.003 | 1.23 (1.06~1.43) | 0.006 | 1.18 (1.00~1.40) | 0.047 |
| TyG tertile |  |  |  |  |  |  |
| T1 (lowest) | Reference |  | Reference |  | Reference |  |
| T2 (middle) | 1.17 (0.88~1.57) | 0.283 | 1.06 (0.80~1.40) | 0.681 | 0.98 (0.75~1.28) | 0.896 |
| T3 (highest) | 1.38 (1.07~1.80) | 0.014 | 1.29 (0.99~1.67) | 0.055 | 1.18 (0.90~1.56) | 0.228 |
| P for trend |  | 0.014 |  | 0.053 |  | 0.212 |
| TyG-WC |  |  |  |  |  |  |
| TyG-WC | 1 (1~1) | <0.001 | 1 (1~1) | <0.001 | 1 (1~1) | 0.009 |
| TyG-WC tertile |  |  |  |  |  |  |
| T1 (lowest) | Reference |  | Reference |  | Reference |  |
| T2 (middle) | 1.13 (0.88~1.46) | 0.348 | 1.07 (0.83~1.36) | 0.614 | 0.98 (0.77~1.25) | 0.856 |
| T3 (highest) | 1.56 (1.15~2.11) | 0.004 | 1.78 (1.33~2.38) | <0.001 | 1.30 (0.95~1.77) | 0.102 |
| P for trend |  | 0.004 |  | <0.001 |  | 0.122 |
| TyG-WHtR |  |  |  |  |  |  |
| TyG-WHtR | 1.34 (1.19~1.50) | <0.001 | 1.51 (1.32~1.73) | <0.001 | 1.34 (1.12~1.60) | 0.002 |
| TyG-WHtR tertile |  |  |  |  |  |  |
| T1 (lowest) | Reference |  | Reference |  | Reference |  |
| T2 (middle) | 1.18 (0.89~1.55) | 0.245 | 1.23 (0.95~1.59) | 0.109 | 1.05 (0.80~1.37) | 0.728 |
| T3 (highest) | 1.65 (1.24~2.19) | <0.001 | 1.85 (1.37~2.50) | <0.001 | 1.33 (0.95~1.85) | 0.095 |
| P for trend |  | <0.001 |  | <0.001 |  | 0.107 |
| TyG-BMI |  |  |  |  |  |  |
| TyG-BMI | 1 (1~1) | 0.274 | 1.01 (1~1.01) | <0.001 | 1 (1~1) | 0.019 |
| TyG-BMI tertile |  |  |  |  |  |  |
| T1 (lowest) | Reference |  | Reference |  | Reference |  |
| T2 (middle) | 0.64 (0.48~0.86) | 0.003 | 1.02 (0.77~1.35) | 0.888 | 0.91 (0.66~1.24) | 0.545 |
| T3 (highest) | 0.82 (0.62~1.09) | 0.171 | 1.82 (1.36~2.43) | <0.001 | 1.35 (0.90~2.01) | 0.145 |
| P for trend |  | 0.156 |  | <0.001 |  | 0.325 |

Model 1: Non-adjusted; Model 2: Adjusted for age, gender, and race; Model 3: Additionally adjusted for marital status, education, PIR, smoking, alcohol use, cancer, CKD, BMI (excluded when analyzing TyG-BMI), total energy intake, TC, LDL-C, BUN, UA, Cr, ALT, AST, ALB, TBil, antidiabetic medication use, statin use, and antihypertensive use.

| Complex survey-weighted multivariable regression analysis between TyG-related indices and the risk of all-cause mortality | | | | | | |
| --- | --- | --- | --- | --- | --- | --- |
| Exposure | Model 1 | | Model 2 | | Model 3 | |
|  | HR(95%CI) | P-value | HR(95%CI) | P-value | HR(95%CI) | P-value |
| TyG |  |  |  |  |  |  |
| TyG | 1.24 (1.14~1.36) | <0.001 | 1.20 (1.09~1.32) | <0.001 | 1.17 (1.06~1.29) | 0.003 |
| TyG tertile |  |  |  |  |  |  |
| T1 (lowest) | Reference |  | Reference |  | Reference |  |
| T2 (middle) | 1.13 (0.96~1.34) | 0.141 | 0.99 (0.84~1.17) | 0.927 | 0.96 (0.82~1.13) | 0.64 |
| T3 (highest) | 1.36 (1.18~1.57) | <0.001 | 1.20 (1.04~1.38) | 0.01 | 1.16 (0.99~1.35) | 0.068 |
| P for trend |  | <0.001 |  | 0.008 |  | 0.054 |
| TyG-WC |  |  |  |  |  |  |
| TyG-WC | 1 (1~1) | <0.001 | 1 (1~1) | <0.001 | 1 (1~1) | <0.001 |
| TyG-WC tertile |  |  |  |  |  |  |
| T1 (lowest) | Reference |  | Reference |  | Reference |  |
| T2 (middle) | 1.08 (0.94~1.23) | 0.305 | 0.99 (0.87~1.14) | 0.927 | 0.95 (0.82~1.11) | 0.533 |
| T3 (highest) | 1.44 (1.24~1.67) | <0.001 | 1.55 (1.34~1.79) | <0.001 | 1.30 (1.09~1.56) | 0.003 |
| P for trend |  | <0.001 |  | <0.001 |  | 0.006 |
| TyG-WHtR |  |  |  |  |  |  |
| TyG-WHtR | 1.30 (1.20~1.40) | <0.001 | 1.41 (1.29~1.54) | <0.001 | 1.36 (1.22~1.53) | <0.001 |
| TyG-WHtR tertile |  |  |  |  |  |  |
| T1 (lowest) | Reference |  | Reference |  | Reference |  |
| T2 (middle) | 1.23 (1.05~1.43) | 0.009 | 1.24 (1.08~1.43) | 0.003 | 1.13 (0.98~1.31) | 0.084 |
| T3 (highest) | 1.59 (1.34~1.87) | <0.001 | 1.68 (1.42~1.99) | <0.001 | 1.41 (1.17~1.71) | <0.001 |
| P for trend |  | <0.001 |  | <0.001 |  | <0.001 |
| TyG-BMI |  |  |  |  |  |  |
| TyG-BMI | 1 (1~1) | 0.027 | 1 (1~1) | <0.001 | 1.01 (1~1.01) | 0.002 |
| TyG-BMI tertile |  |  |  |  |  |  |
| T1 (lowest) | Reference |  | Reference |  | Reference |  |
| T2 (middle) | 0.69 (0.59~0.80) | <0.001 | 1.01 (0.87~1.16) | 0.942 | 0.97 (0.84~1.13) | 0.729 |
| T3 (highest) | 0.76 (0.64~0.91) | 0.003 | 1.46 (1.23~1.74) | <0.001 | 1.24 (0.99~1.54) | 0.056 |
| P for trend |  | 0.002 |  | <0.001 |  | 0.145 |

Model 1: Non-adjusted; Model 2: Adjusted for age, gender, and race; Model 3: Additionally adjusted for marital status, education, PIR, smoking, alcohol use, cancer, CKD, BMI (excluded when analyzing TyG-BMI), total energy intake, TC, LDL-C, BUN, UA, Cr, ALT, AST, ALB, TBil, antidiabetic medication use, statin use, and antihypertensive use.
