# Supplementary material for: Evaluative performance of TyG-ABSI versus traditional indices in relation to cardiovascular disease and mortality: evidence from the U.S. NHANES
Source: Cardiovasc Diabetol. 2025 Aug 21;24:344. doi: 10.1186/s12933-025-02902-6 (PMC12372269; doi:10.1186/s12933-025-02902-6)
Supplement: Supplementary file 4 — Supplementary Material 4 [file 12933_2025_2902_MOESM4_ESM.docx]

AUC values of TyG-related indices for predicting CVD

| **Variable** | **AUC (%)** | **95% CI (%)** |
| --- | --- | --- |
| TyG | 55.28 | 53.78 – 56.78 |
| TyG-ABSI | 62.22 | 60.79 – 63.66 |
| TyG-WC | 55.16 | 53.67 – 56.65 |
| TyG-WHtR | 55.43 | 53.94 – 56.93 |
| TyG-BMI | 52.54 | 50.99 – 54.10 |

AUC values of TyG-related indices for predicting cardiovascular mortality

| **Variable** | **AUC (%)** | **95% CI (%)** |
| --- | --- | --- |
| TyG | 53.55 | 51.13 – 55.97 |
| TyG-ABSI | 63.46 | 61.21 – 65.71 |
| TyG-WC | 52.07 | 49.65 – 54.48 |
| TyG-WHtR | 51.82 | 49.38 – 54.26 |
| TyG-BMI | 58.28 | 55.72 – 60.84 |

AUC values of TyG-related indices for predicting all-cause mortality

| **Variable** | **AUC (%)** | **95% CI (%)** |
| --- | --- | --- |
| TyG | 53.22 | 51.78 – 54.66 |
| TyG-ABSI | 64.12 | 62.77 – 65.47 |
| TyG-WC | 52.12 | 50.68 – 53.56 |
| TyG-WHtR | 51.69 | 50.25 – 53.13 |
| TyG-BMI | 59.03 | 57.54 – 60.51 |
